# Timing of Circulatory and Neurological Events in Syncope

**DOI:** 10.3389/fcvm.2020.00036

**Published:** 2020-03-13

**Authors:** J. Gert van Dijk, Ineke A. van Rossum, Roland D. Thijs

**Affiliations:** ^1^Department of Neurology, Leiden University Medical Centre, Leiden, Netherlands; ^2^Stichting Epilepsie Instellingen Nederland, Heemstede, Netherlands

**Keywords:** syncope, tilt table test, vasovagal syncope, transient loss of consciousness, pathophysiology

## Abstract

Syncope usually lasts less than a minute, in which short time arterial blood pressure temporarily falls enough to decrease brain perfusion so much that loss of consciousness ensues. Blood pressure decreases quickest when the heart suddenly stops pumping, which happens in arrhythmia and in severe cardioinhibitory reflex syncope. Loss of consciousness starts about 8 s after the last heart beat and circulatory standstill occurs after 10–15 s. A much slower blood pressure decrease can occur in syncope due to orthostatic hypotension Standing blood pressure can then stabilize at low values often causing more subtle signs (i.e., inability to act) but often not low enough to cause loss of consciousness. Cerebral autoregulation attempts to keep cerebral blood flow constant when blood pressure decreases. In reflex syncope both the quick blood pressure decrease and its low absolute value mean that cerebral autoregulation cannot prevent syncope. It has more protective value in orthostatic hypotension. Neurological signs are related to the severity and timing of cerebral hypoperfusion. Several unanswered pathophysiological questions with possible clinical implications are identified.

## Introduction

Two key features of syncope are that is a form of transient loss of consciousness and that it is due to cerebral hypoperfusion (1, 2). The cerebral hypoperfusion in syncope is global in nature, so it may affect all brain functions, though not necessarily always or all at once. This global cerebral hypoperfusion stems from a failure of the systemic circulation to keep arterial blood pressure high enough to perfuse the brain adequately.

This paper provides an overview of the timing of cardiovascular and neurological events in syncope. The discussion of reflex syncope will address some general aspects that apply to all forms of syncope. These will not be repeated in the discussion of cardiac syncope and syncope due to orthostatic hypotension. Some unanswered questions will be identified.

## Reflex Syncope

### The Reflex Pathway

Transient loss of consciousness (TLOC) in syncope is the result of a temporary low blood pressure. As arterial blood pressure (BP) is the product of cardiac output and total peripheral resistance (TPR), and as cardiac output is the product of stroke volume (SV) and heart rate (HR), BP is the product of all three: BP = SV·HR·TPR. This simple equation means that a decrease of any one of these three parameters can in principle cause syncope, provided the decrease is severe enough, lasts long enough and is not compensated for sufficiently by an increase of any of the other parameters.

The terms “reflex syncope” and its synonym “neurally mediated syncope” indicate that syncope is evoked by a trigger setting afferent nerve impulses in motion. However, reflex syncope does not only involve the nervous system. Hormonal factors are likely to play a role as well. There are obvious similarities between neuronal reflex and endocrinological mechanisms of regulation: both involve the detection of a disturbance and a corrective response. The sections below focus primarily on autonomic nervous system responses, and provide a short overview of humoral influences.

The array of possible triggers in reflex syncope comprises extremely diverse elements; examples are thinking unpleasant thoughts, eating a heavy meal, stretching, stopping with running, coughing, being stuck with a needle, or simply standing still. It is hard to understand why such diverse impulses would all evoke a very similar response, and yet they do. Clinical experience suggests reflex syncope is more likely to occur when several risk factors occur at the same time. It seems likely that a low circulating volume or an already low blood pressure feature in this set of common factors, but it is unknown whether any one factor is obligatory in triggering reflex syncope.

The neuronal efferent responses in reflex syncope can be identified with more confidence because their specific efferent circulatory effects are also involved in normal baroreceptor blood pressure control. The nuclei transmitting the efferent impulses are the dorsal vagal nucleus and the nucleus tractus solitarii. These control two effector mechanisms that are normally involved in blood pressure control. In reflex syncope these two mechanisms can both lower blood pressure: they are the cardioinhibitory (CI) and vasodepressive (VD) pathways.

#### Cardioinhibition

CI concerns an increase of vagal stimulation of the heart, resulting in a decrease of HR. The severity of CI ranges from a slight HR decrease to long asystole. Asystole during tilt table testing (TTT) may last about 1 min, but that is not the typical duration. Asystole, defined as a pause of at least 3 s. duration, occurred in 21 of 69 TTT cases (30.4%) selected for the occurrence of full syncope; the mean duration of asystole was 9.1 ± 6.4 s (3). The duration and asystole rate in TTT depend on test characteristics such as the duration of tilting back (4). Note that the inhibitory effects of CI not only concern the sinoatrial (SA) node affecting HR, but also the atrioventricular (AV) node: in tilt-induced VVS, the occurrence of AV escape beats suggests that the AV node is less inhibited than the SA node (5). In contrast, a complete AV-block, also occurring in VVS, suggests stronger inhibition of the AV than the SA node. These observations in turn strongly suggest that the common presentation of asystole without any electrical heart activity is due to complete inhibition of both nodes.

A decrease of HR can lead to a quick decrease of BP, as is evident from the sudden cardiac standstill that occurs in complete AV-block. BP may then be halved in just 3 s. after the last beat; after about 10–15 s. of persistent asystole, BP becomes stable at 10–20 mmHg (6). At this extremely low BP, there is no longer a pressure difference between the aorta and right ventricle, so systemic blood flow stops altogether. The available evidence suggests that loss of consciousness starts about 8 s. after the last heart beat (6, 7). Patients are typically not aware of anything amiss for the first 3 of 4 s. of that time; those who do sense something amiss have only a few s. to lie down before consciousness is lost and they fall.

#### Vasodepression

Vasodepression (VD) can be interpreted as any mechanism in reflex syncope apart from CI that causes blood pressure to decrease. As explained below, a decrease in venous return is one such cause. The other is a decrease of sympathetic vasoconstriction of arterioles, and some authors restrict VD to this mechanism. This decrease allows arterioles to dilate, resulting in a decrease of Total Peripheral Resistance (TPR). The shortest time in which sympathetic effects can affect BP through this mechanism is probably a few heart beats, whereas vagal effects on HR can operate within one heartbeat.

#### CI and VD in Reflex Syncope

In reflex syncope, VD and CI may occur together to varying degrees, making it difficult to determine how much either contributes to the decrease of BP. Even so, it is safe assumed that asystole, if it lasts long enough, will always cause the circulation to come to a halt and cause syncope; this will occur irrespective of any additional VD.

The nature and severity of CI differs between types of reflex syncope. In swallow syncope asystole due to an AV-block has been frequently described (8), although a selection bias cannot be ruled out. This “reflex AV-block” differs in some respects from an intrinsic AV-block, and is probably due to stronger inhibition of the AV- than the SA-node. Age plays a part too, as asystole in VVS occurs more often in younger patients than in older ones. This is most obvious in the so-called “pallid breath holding spells” of toddlers, in which bradycardia and asystole cause LOC (9). Note that these sadly misnamed spells do not involve abnormal breathing, in contrast to “cyanotic breath holding spells” (10). The “pallid” spells are in fact nothing other than cardioinhibitory VVS (1). In adults with VVS, CI occurs more in younger people than in elderly ones (11–13).

Over the last 15–20 years the concept that reflex syncope involves CI and VD in the shape of reduced arteriolar vasoconstriction had to be revised substantially for VVS. As these studies mostly concerned TTT, the results hold for VVS induced by standing (“orthostatic VVS”). The existence of CI remained undisputable in view of obvious bradycardia and asystole. But a decrease of TPR was not consistently present. In many of these studies Modelflow was used, a technique relying on analysis of the arterial pressure waveform of individual heart beats (14). TPR did not consistently decrease before syncope: although a decrease in TPR was reported in young people (15), an increase was been described too (15). Moreover, TPR could increase before syncope in some patients but not in others (16). Finally, during actual syncope TPR even proved to be increased rather than decreased in adults (17–19) and children (20). The increase of TPR at syncope in these Modelflow studies seems to contradict studies using another technique, “muscle nerve sympathetic activity” (MSNA). The latter technique records sympathetic vasoconstrictor nerve impulses to blood vessels in leg muscles. Most MSNA studies show a cessation of such impulses during syncope (21, 22), but this does not occur in all cases (23, 24). There is therefore an apparent discrepancy between Modelflow and MSNA techniques. Solving this probably require a simultaneous assessment of clinical characteristics of syncope, Modelflow and MSNA.

Instead of the expected decrease in TPR, a decrease in SV emerged as a consistent finding in the minutes before syncope in tilt-induced VVS (15, 18, 25–28). This low SV was usually attributed to a decrease of venous return, in turn due to venous pooling in the lower limbs, abdomen or pelvis (15, 18, 28–30). The current concept of the circulatory events in the minutes before VVS holds that venous pooling causes a decrease in SV that in turn leads to a slow decrease in BP. CI occurs much later in the course of this process, but not in all cases. CI may well be triggered by the ongoing venous pooling and resulting decrease in blood pressure. Whether VD must be present for CI to occur is uncertain: findings in toddlers suggest that CI can occur without antecedent VD (9).

While it has become clear that venous pooling acts as the starting point of orthostatic VVS, it is not clear why this occurs at some times, and its place in the “reflex” concept is not clear. It may be seen as the triggering process rather than as part of the response. If it is a trigger, then the neuronal reflex may in fact only become active at the moment when CI starts. Whether the reflex then also has a component of a reduction of arteriolar vasoconstriction (low TPR), is not yet clear.

At any rate the question may be asked whether the term “vasodepression” should be limited to a sympathetically mediated release of arteriolar vasoconstriction. A wider concept of VD, comprising both arterial and venous components, has already been proposed (31). The arteriolar component of VD would then appear as a decrease of TPR, while the venous one is reflected in a decrease of SV.

#### The Timing and Importance of CI in Reflex Syncope

In tilt-induced VVS, BP may start to decrease several minutes before syncope, while CI typically starts later, a minute or less before syncope. As said, CI may be triggered by a secondary abnormality related to the ongoing venous pooling. Putative secondary triggers may be sought in consequences of venous pooling, such as a reduction of blood volume in the thorax, likely to alter the filling, flow or pressure in thoracic blood vessels and in the heart itself. The “empty heart hypothesis” sought to explain the origin of CI in mechanoreceptors in the walls of the near-empty ventricles (32); however, evidence against this theory was later reported (33, 34). Over 20 years later, what triggers CI in tilt-induced VVS still remains unknown.

It should be understood that a lowering of HR at a time when BP is decreasing contradicts the normal behavior of the baroreflex. This reflex normally aims to keep BP constant by manipulating HR as well as TPR. Normally a lowering of BP elicits a rise in HR, and a BP increase will evoke a HR decrease. After CI sets in, BP and HR decrease together, showing that normal baroreflex control is lost (35). Baroreceptor control is in fact already abnormal earlier, when subjects became symptomatic (36).

While tilt-induced VVS appears to be a good model for orthostatic VVS (37), its findings need not apply to other forms of reflex syncope. Which roles the three factors CI, arterial and venous VD, play in emotional VVS and other forms of reflex syncope such as carotid sinus syncope remains to be studied. However, the differing rates of CI between forms of reflex syncope provide evidence that CI can systematically differ in strength between forms. The relative contributions of arterial and venous VD probably also differ: for instance, massage of a carotid artery can result in syncope in such a short time that a slow build-up of venous pooling is very unlikely. Unraveling the relative size and timing of these three effects is likely to have practical consequences, as this may impact pacemaker therapy.

CI and VD can each cause syncope on their own: VD can cause LOC in the absence of any decrease of HR (7), and can even cause EEG flattening (see below) as evidence of profound cerebral hypoperfusion (3). Asystole, if long enough, will cause LOC regardless of any degree of VD. Unfortunately, a consequence of the occurrence of simultaneous VD and CI is that preventing or shortening asystole need not prevent syncope. Given that VD can cause syncope on its own, and that CI and VD often occur together, a pacemaker is then not a failsafe way to prevent syncope.

The relative strength of CI and VD is not the only important factor in determining whether syncope will occur; their time of onset also determines the outcome (31). The timing of CI was studied in cases of tilt-induced syncope, documented with continuous blood pressure monitoring as well as video-EEG, excluding cases with presyncope only or the use of nitroglycerin. In one third of the resulting 35 cases, asystole started either after the onset of LOC or <3 s. before it, short enough to mean that asystole could not have caused LOC that quickly. In those with late asystole, the median BP at the onset of asystole was 32.0 vs. 45.5 mmHg in those with early asystole. This suggested that VD had already lowered BP severely in those with late asystole. These results help explain some of the failures of pacemakers to prevent syncope, and strongly suggest that relying on HR alone is likely to overestimate the importance of asystole as the cause of syncope (31).

### Humoral Influences

For a detailed review of the possible roles of neurohormones in VVS we refer to Benditt et al. (38). Here, a very short summary must suffice. The best known alterations of hormones in syncope concern adrenalin and noradrenalin in orthostatic VVS. A general finding during presyncope is that the ratio of adrenalin to noradrenalin rises, due to a large increase of adrenalin and a very limited or no increase of noradrenalin (38). Higher ratios were linked to a shorter time to syncope (39, 40). Adrenalin may have different vasoconstrictor effects on different vascular beds, while noradrenalin is a potent vasoconstrictor. The high adrenalin to noradrenalin ratio may therefore induce vasodilation is some vascular beds. As such, this hormonal shift might contribute to the venous pooling observed in VVS. It probably does not explain most of the pooling, as adrenergic blockers did not significantly prevent VVS (41).

Various other hormones and circulating agents have been investigated, such as vasopressin and natriuretic peptides. At present it remains to be seen whether hormonal alterations are a response to a circulation that is slipping out of autonomic control, or whether they contribute to it (38).

### Cerebral Perfusion in Reflex Syncope

**Figure 1:**
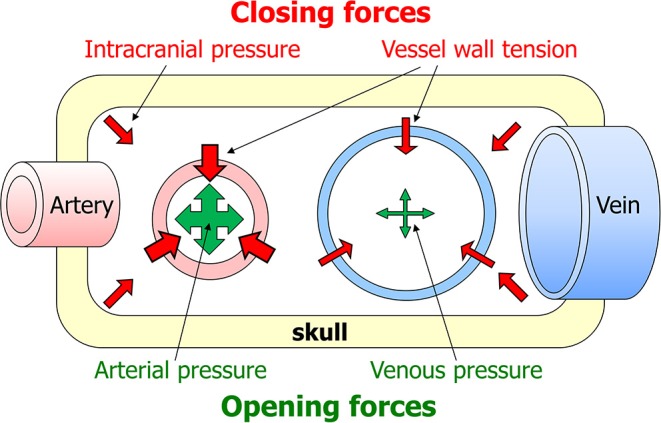
Factors affecting cerebral blood flow. Blood will only flow through the brain if two criteria are met: there must be a pressure difference between the arteries and veins supplying the brain, and the vessels in between must be open. The latter depends on a summation of forces opening and closing the vessels. The main opening force is the pressure in the lumen of the vessel. The two closing forces are intracranial pressure and vessel wall tension.

Blood will only flow through an organ if two conditions are met: firstly, there must be a pressure difference between the arteries and veins supplying the organ. Secondly, the vessels in between must be open (42). For flaccid vessels such as veins, the main force that keeps them open is intraluminal pressure. These simple principles suggest that two aspects can critically lower organ perfusion. The first is that veins are more likely to be occluded than arteries, as their intraluminar pressure is lower. Secondly, the phase of the heart beat is important, as intraluminar pressure is lower during diasystole than systole. Blood flow through organs is generally lower during diastole than systole, but the brain is exceptional in having a substantial diastolic flow (43). The combined result of these effects is that perfusion is most at risk in veins and during diastole. Arrayed against intraluminal pressure as the one force opening vessels are two closing forces: vessel wall tension and tissue pressure. In the brain, tissue pressure equals intracranial pressure (Figure 1). As venous and intracranial pressures are normally very low, arterial pressure normally is the predominant force in cerebral perfusion.

The comparison of opening with closing forces helps explain brain perfusion in abnormal conditions. A surprising consequence of this is that cerebral flow in imminent brain death resembles that in syncope. In brain death intracranial pressure is so high that the closing forces gain the upper hand. A reflex increase of systemic blood pressure counters the high intracranial pressure in part, but beyond a certain point intracranial pressure will approach blood pressure. Transcranial Doppler (TCD) then shows that diastolic flow velocity decreases before systolic velocity does; this is because the opening forces are at their weakest in diastole. In syncope BP is so low that the closing forces again win, but now because the opening forces are very weak. Again, TCD shows that systolic flow velocity remains nearly normal while diastolic velocity is markedly reduced.

### Cerebral Autoregulation in Reflex Syncope

Cerebral autoregulation describes the ability of brain perfusion to counteract changes in arterial BP: when BP decreases, the forces that close vessels decrease to keep cerebral perfusion constant. Likewise, when BP increases, vessels must constrict to increase the closing forces in parallel with the increase in the opening force. Cerebral autoregulation has a lower limit of regulation of about 60 mmHg. If B falls below this level, cerebral perfusion will decrease in parallel with BP (43). Cerebral autoregulation depends on endothelial, myogenic metabolic and neurogenic factors. It is considered to have static and dynamic components; the latter one acts on a time scale of 2 to 10 s (44).

The time scale of autoregulation is critical for reflex syncope. In case of a sudden asystole, BP will fall below the lower level of autoregulation before that autoregulation has had the time to exert its full effect. When BP decreases slowly, as may happen with pure VD, autoregulation has more time to counteract the effects of decreasing BP. An elegant study found that cerebral resistance to flow was markedly reduced at syncope, meaning that autoregulation indeed opened vessels to compensate for low BP (45), confirmed later (46, 47). These saving attempts are likely to fail in the end, as the decrease of BP in reflex syncope not only continues but typically even accelerates (1, 7, 35). Both an overly quick BP decrease and a very low BP will therefore eventually overcome the compensatory effects of autoregulation.

### The Clinical Characteristics of Reflex Syncope

Not all symptoms and signs associated with syncope are due to cerebral hypoperfusion (6). Some symptoms instead reflect the cause of syncope, which can be very useful for diagnosis. For instance, syncope preceded by palpitations and chest pain points strongly to cardiac syncope, while the prodromes of nausea, sweating and facial pallor strongly suggest a reflex mechanism. Although the latter are sometimes described as “autonomic activation,” they need not all depend on the autonomic nervous system. Humoral factors may well play a role. Further discussion will be limited to signs of cerebral hypoperfusion.

#### Signs of Cerebral Hypoperfusion

**Figure 2:**
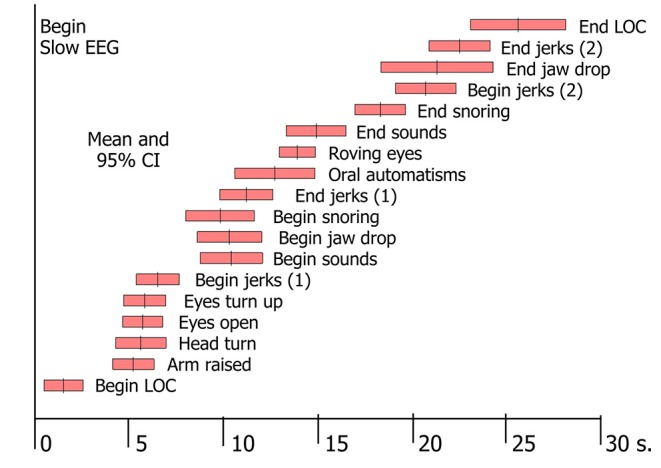
Signs in tilt-induced VVS. Each bar represents the mean value and the 95% confidence intervals for a range of signs, determined by analysis of video-EEG records of 69 cases of tilt-induced VVS [These data conform to Table 2 in Van Dijk et al. (3)].

In tilt-induced VVS, the first observable signs of LOC were motor ones, i.e., a vacant expression or dropping of the head and jaw (3). From that point on, a large number of phenomena could be witnessed (3, 48). A specific order of progression was observed, although the time of onset of signs varied between individuals [(3); Figure 2]. The signs proved to be linked to the two EEG patterns of syncope: with mild hypoperfusion the EEG shows slowing (“S”) only, whereas more severe hypoperfusion results in a slow-flat-slow pattern (“SFS”) (3, 49). The EEG concerns cortical functions only, so any conclusions regarding basal ganglia or brainstem dysfunction are wholly based on clinical observations. Brainstem signs appeared later than cortical ones, because the brainstem is more resistant to ischaemia. Some signs were strongly associated with EEG flattening, and are therefore likely to concern brainstem activity: making sounds, snoring, upward deviation of the eyes and roving eye movements (3). We later added tonic posturing of the arms to this list (48), either flexion or extension. As more severe cerebral hypoperfusion was associated with lower minimal BP and asystole (3), these signs should increase suspicion of deep hypoperfusion, in turn likely due to CI or arrhythmia.

**Figure 3:**
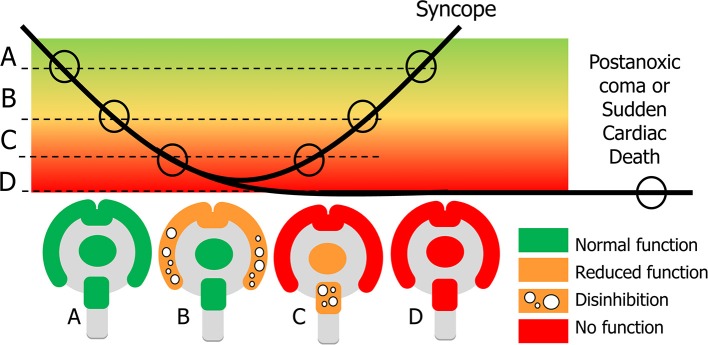
Explanation of signs in syncope and sudden cardiac death. In syncope cerebral perfusion (bold line) temporarily decreases below the normal level **(A)**. Clinical signs depend on the severity of cerebral hypoperfusion. Dysfunction becomes apparent in two ways: normal function can be decreased or completely lost, and new actions may appear, caused by neuronal disinhibition. With mild hypoperfusion this occurs for the cortex, while the basal ganglia and brainstem still function normally: state **(B)** appears at the beginning and end of syncope. With more severe hypoperfusion all cortical function is lost, and new signs concern a decrease of function and disinhibition of the brainstem **(C)**. If recovery takes too long, there will be cerebral damage such as the postanoxic coma; if there is no circulatory recovery at all, the result is sudden cardiac death **(D)**.

Some syncopal signs indicate a loss of neuronal function whereas others can only be interpreted as abnormal excessive neuronal activity, probably due to a lack of inhibition. Examples of the latter are mumbling, pointing to cortical disinhibition, and roving eye movements, suggesting brainstem disinhibition (3). Both the cortex and the brainstem appear to go through a sequence in which a loss of some activity and disinhibition happen first, followed by a complete loss of activity [(48), Figure 3].

### Cardiac Syncope

“Cardiac syncope” is usually taken to comprise both specific cardiac causes as well as disorders of the great vessels, such as pulmonary embolism (1). The two main groups are arrhythmia and structural heart disease. An arrhythmia concerns an abnormal rhythm of heart beats, consisting of irregular beats, too fast beats (tachycardia), and too slow beats (bradycardia). Arrhythmias may be permanent or paroxysmal. Syncope in arrhythmia is usually die to a sudden onset of an arrhythmia, leading to an abrupt decrease of cardiac output and hence of blood pressure. In bradycardic arrhythmia, low HR causes cardiac output to fall. Bradycardia with HR below 30 bpm for 15–30 s. cause syncope more often than tachyarrhythmias. In tachycardia, there is too little time to fill the heart in diastole, so SV falls precipitously, more than is accounted for by the high HR. Arrhythmias often occur without a recognizable trigger, although some occur during exercise (see below). Arrhythmias generally occur regardless of posture. This is important clinically: forms of syncope that primarily depend on non-cardiac BP abnormalities tend to occur more readily while standing, and sitting or lying down helps prevent syncope. But if the circulation stops altogether, syncope occurs regardless of posture. This explains why syncope in the supine position is a danger sign for cardiac syncope.

Structural heart disease concerns heart diseases, other than coronary heart disease, of a mechanical nature, including valvular disease, septum defects, and cardiomyopathy. Structural heart disease impairs the mechanical efficiency of the heart. This becomes most problematic when there is a discrepancy between the demands for a high cardiac output and a failure to deliver it. This helps explains why syncope in relation to exercise is a cardiac danger signs. However, if low cardiac output were the only mechanism, patients might be expected to experience a slow inability to perform physical work. Instead, the typical history is a sudden LOC with little or no warning. The explanation for the latter pattern may be a susceptibility to respond to hypotension with syncope, so the mechanism in structural cardiac syncope may in fact partly be reflex syncope (7, 37, 50).

In many cases cardiac syncope develops very quickly, resulting in the same clinical signs as in sudden complete CI in reflex syncope: patients may only have a few s. to become aware of a problem before they lose consciousness. But the situation may be more complicated when the pumping action is not lost entirely, so cardiac output is decreased but not nil. We have noted patients becoming aware of “weakening” or “dizziness” without LOC, even in cases of ventricular tachycardia.

### Sudden Cardiac Death

The term “convulsive syncope” appears to be used to indicate a perceived high severity of syncope, and suggests that it is based on the presence of myoclonic jerks. However, jerking of the arms did not occur significantly more often with severe cerebral hypoperfusion, i.e., EEG flattening, then without such flattening (3), so there is no evidence that the presence of myoclonic jerks in syncope indicates severe hypoperfusion. However, it arrhythmic syncope might last much longer than tilt-induced syncope, and it is possible that extremely long-lasting syncope causes neurological signs that are not observed during TTT. Unfortunately, there appear to be no reports on the clinical signs of such long syncope. Personal communications from physicians familiar with resuscitation in emergency rooms (S Peeters) or the intensive care (J Maas) do not suggest that recovery from long-lasting cardiac standstill is accompanied by dramatic abnormal movements. In contrast, movements then appear to be absent altogether.

There are recent observations of sudden cardiac death, taken from publicly available video records of sports players (51–55). One report mentioned that all six observed players had their eyes wide open with a fixed gaze and fixed pupils (55). This led the authors to conclude that a sudden unexpected LOC in an athlete in action and a fixed gaze were key features of sudden cardiac death. As these signs do not differ from those in severe tilt-induced VVS (3), we suggest that they in fact point to severe cerebral ischaemia, regardless of cause. It is the occurrence of these signs in relation to exercise is the tell-tale feature that should raise suspicion of a potentially deadly cause. It is also noteworthy that most observed cases of sudden cardiac death during sports did not occur during intensive action, but shortly after it or during non-intensive action (52, 53). Hence, syncope just after cessation of exercise may not be safely assumed to be due to VVS or orthostatic hypotension.

## Syncope Due to Orthostatic Hypotension

There are three types of orthostatic hypotension (OH); the initial, classical and delayed types: iOH, cOH, and dOH (1). Note that the definition of cOH conforms to previous definitions of “OH”. The distinction between types of OH became necessary because of the growing recognition of iOH and dOH as separate entities. To avoid using the same term “OH” for the entire group as well as for one of its parts, “OH” now is an umbrella term. The discussion will center on cOH; for iOH and dOH we refer to other sources (56–58).

### Definitions and Causes of cOH

The definition of cOH (1, 59) describes a sustained fall of at least 20 mmHg for systolic BP, or of at least 10 mmHg for diastolic BP, within 3 min of standing up or of being tilted upwards. The ESC criteria added an absolute systolic BP of <90 mmHg (1). In daily practice the systolic criterion may have more value than the diastolic one (60). Note that the definition does not state whether or not the BP decrease should be accompanied by symptoms. It merely describes a measurement result that points to a failure to keep BP at the desired level in the upright position.

The causes of cOH are a failure to control arteriolar vasoconstriction, hypovolemia or both. The most common cause of cOH is medication. When the cause concerns structural damage to the sympathetic nervous system, the resulting cOH is often labeled “neurogenic OH” [nOH; (61)]. The damage may be a secondary effect of nerve damage, as in diabetes, amyloidosis, or auto-immune autonomic neuropathy, or it may concern primary autonomic failure, in which the autonomic nervous system suffers primary damage. Primary autonomic failure causing cOH consists of pure autonomic failure (PAF), multiple system atrophy (MSA), Parkinson's disease with OH (PD-OH), and Lewy body dementia (LBD).

### Pathophysiology of cOH/nOH

The central defect of nOH is an inability to bring about arteriolar vasoconstriction (62). In terms of the factors governing BP, i.e., HR, SV, and TPR, this concerns an inability to increase TPR when needed. To understands its effects the normal events in standing up must be understood: standing up is associated with a fall in SV due to a downwards movement of blood. The normal physiological response to this consists of compensatory increases in HR and TPR. The overall effect is usually an increase in diastolic BP and a slight increase or no change in systolic BP. As said, the prime abnormal event in cOH is a failure of TPR to increase in the upright position, but the situation is more complicated: the failure of arterial vasoconstriction causes a secondary venous pooling, perhaps exacerbated by a failure to control the volume in the venous vascular beds (62). This venous pooling will become apparent as a low SV, further decreasing BP. The only remaining mechanism to repair BP would be an increase of HR, but damage to the autonomic nervous system may also involve an inability to control HR adequately. If so, neither HR and TPR can rise, while SV is low.

BP measurements in patients with cOH/nOH exhibit two features that point to even more complicated processes: supine hypertension (63) and a BP overshoot when patients lie down or are tilted back after head-up tilt (64). Supine hypertension may be present at every occasion a patient lies down during the day, and may also be present throughout the night. Its existence suggests that attempts to compensate for low upright BP keep on functioning for hours in the supine position. The BP overshoot after head-up tilt also suggests a tendency toward a high pressure, and shows that BP can in fact increase very quickly, gaining as much as 60 mmHg within 1 or 2 min. These observations mean that the pathophysiology of cOH is not straightforward and that the primary defect may appear buried under secondary abnormalities.

One unexpected finding in nOH was a decrease of TPR in the upright position, measured from a baseline supine position (64–67). This upright decrease in TPR, apparently stronger in PAF than in MSA, was unexpected because a change to the upright position should cause TPR to increase, or at least remain the same if it cannot increase. Hence, the decrease of TPR requires an additional explanation. One was that the TPR calculations might be incorrect (68), which would however not explain why there were differences between MSA and PAF (64, 66), even when both groups had similar BP falls (64). Another explanation is that there are additional vasodilatory influences that only become active while standing. In physical exertion and indeed active standing a degree of muscular vasodilation occurs that is normally counteracted by the autonomic system, but not if that is damaged (69). A second possibility concerns hyperventilation; this normally causes peripheral vasodilation, but this is again normally countered by the autonomic nervous system. When that is damaged, the vasodilation of hyperventilation may remain unchecked (70, 71).

### Cerebral Autoregulation in OH

Patients with nOH may be fully conscious at remarkably low BP values, suggesting excellent autoregulation (72). Cerebral autoregulation depends on some factors that do and some that do not involve the autonomic nervous system, so the question arises whether autonomic damage can also compromise autoregulation. One approach reasons that good autoregulation would mean that cerebral flow would stay stable even if BP varies; its dysfunction would mean that cerebral blood flow would follow fluctuations in BP. Studies along these lines were conflicting: some pointed to defective autoregulation (65, 73), whereas others showed good function (72, 74–77). Whether the differences depend on the cause of autonomic failure has not been elucidated.

### Signs in OH

As said, signs and symptoms in syncope may either be related to the cause of syncope, or to cerebral perfusion. In nOH, examples related to the cause of syncope include the absence of “autonomic activation” as occurs in reflex syncope. Another example is the occurrence of complaints in the standing position, often exacerbated during exercise or just after it (78, 79). Some patients will report an immediate worsening of symptoms on raising the arms above the head, effectively meaning that the arms compete with the brain for blood flow. Another example is “coat hanger pain,” i.e., pain in the neck and shoulders in the upright position, relieved quickly by lying down (80). It is attributed to local muscle ischaemia.

**Figure 4:**
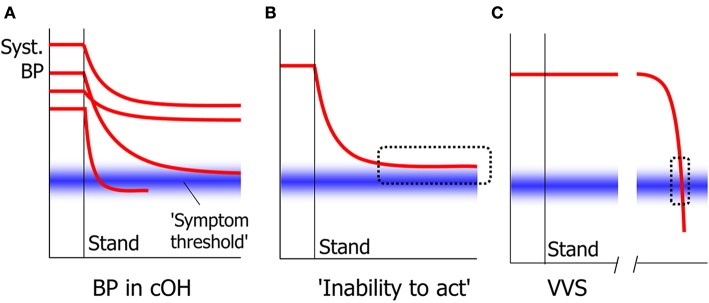
Signs in slow and quick cerebral hypoperfusion. In cOH, BP decreases after standing up to a varying degree and with varying speed in different people **(A)**. The general tendency is for BP to decrease quickly at first and slower later. Whether symptoms and signs appear depend on whether a symptom threshold of cerebral perfusion is crossed. One result of this is that BP may stabilize at a level too high for syncope to occur, yet too low for patients to act volitionally, causing an “inability to act” **(B)**. In VVS, BP decreases slowly at first, but accelerates thereafter. This means that the critical level at which symptoms such as the inability to act occur, is crossed very quickly that this phase is usually not noted **(C)**.

The signs of cerebral hypoperfusion in syncope are in principle irrespective of the cause. Whether they are perceived and reported depends critically on the speed of the blood pressure decrease. When this is very fast, patients often report either no awareness of anything amiss at all, or a non-descript yet recognizable feeling for a few seconds. In cOH, the BP decrease can be quick initially, but the rate of decrease typically diminishes and BP tends to become stable at a lower level. If that level is below that needed to maintain consciousness, syncope ensues; if it is higher, there may be no symptoms or signs. Because BP decreases slowly in cOH, most patients learn to heed the warning signs and learn to prevent syncope. One sign occurs fairly often in nOH: an “inability to act.” In nOH, BP is then too high to cause unconsciousness, but too low to allow higher cortical functions such as deciding to sit down (Figure 4) (81). The result is that patients freeze in their position, either sitting or standing, and may not act until someone pulls them on a chair or a bed. We suspect that this condition in nOH is often mistaken for motor problems or for sleep. This inability to act can in fact also occur in quickly developing syncope, but then only lasts a few seconds (82).

## Conclusions and Future Research

### Conclusions

The above discussion shows that the speed and timing of circulatory and neurological events is of prime importance to understand the signs and symptoms of syncope. Syncope concerns a change from fully functional alertness and awareness to, in the most severe forms, one in which cortical functions have stopped altogether while the brainstem is on the way to equal that state. The quickest natural way to reach that state is probably a sudden asystole, leading to LOC in about 8 s., in which patients may only realize that something was wrong when they regain consciousness after the fact. At the other end of the spectrum lies the slow BP decrease in nOH, in which patients may perceive a slow progression of symptoms over minutes, in which an inability to rectify the situation may precede a loss of vision and finally LOC. Important factors in the equation are the occurrence of bradycardia or asystole on the cardiovascular side, and cerebral autoregulation on the cerebral end.

The timing and speed not only have consequences for the clinical presentation of syncope, but also for therapy. Whether attempts to rectify CI with a pacemaker will prevent syncope in reflex syncope will critically depend on simultaneous VD. The fact that CI started too late to cause LOC in one third of cases with tilt-induced reflex syncope, suggests that pacing will fail at least that same rate, if started at the onset of asystole (31).

### Gaps in Knowledge and Possible Solutions

The above overview helps delineate a number of gaps in the understanding of the processes that lead to LOC in reflex syncope.

The first is limited knowledge concerning the pathophysiology of emotional VVS and other forms of syncope. The reason is very likely that orthostatic VVS can be evoked with relative ease with a TTT, while the other forms, in particular emotional VVS, are difficult to evoke. TTT studies on VVS have clarified that the original scheme of a dual parasympathetic and sympathetic reflex action, respectively causing bradycardia and too little arteriolar vasoconstriction, is at best incomplete. Instead, venous pooling causing a reduction in SV seems to be the prime abnormality starting the complex process of orthostatic VVS. As other forms of reflex syncope are triggered much swifter, these may not rely on the slow process of venous pooling. Hence, studying other forms of reflex syncope may clarify pathophysiological differences between the various types.

A second problem is why and how excessive venous pooling occurs in orthostatic VVS. This reflects an excessive shift of blood from the thorax to lower regions of the body. At present the assessment of venous pooling relies mostly on indirect deductions of its presence from a reduction in SV, i.e., it relies on measurements that concern the arterial system. Instruments that would allow direct measurements of venous filling or the distribution of blood across body regions might yield as much new information regarding the venous system as continuous blood pressure measurements did for the arterial system. Impedance measurements should help fill his gap, and have shown important results, but are not used widely (29, 83).

A third and fundamental problem is why the autonomic nervous system seems incapable of countering venous pooling in orthostatic VVS. The reduction in SV leads to a slow decrease in BP, meaning that this decrease is for some reason not countered by adequate increases in sympathetic arteriolar vasoconstriction nor heart rate. Are there conflicting demands on the autonomic nervous system that prevent it from acting normally, or is there perhaps a mismatch between humoral and nervous actions?

Fourth, it is difficult to assess which of the processes CI and VD affect BP the most when both act together. In the well-known equation BP = SV·HR·TPR, HR represents CI, and SV and TPR may represent venous and arterial VD. The multiplicative nature of this relation suggests that it should be possible to determine how much each parameter contributes to a given decrease in BP. If such comparisons would for instance reveal that over half of a BP decrease is due to a HR decrease, pacing attempts become more promising then when CI hardly explains any BP decrease. Clearly, not only the relative severity of VD and CI, but also their relative time of onset is important for the occurrence of syncope: in about one third of cases of tilt-induced VVS, asystole occurred too late to cause syncope (31). Treatment decisions regarding pacing, at least in orthostatic VVS, should therefore ideally not be based on ECG records only. Measuring VD at the same time would be helpful, provided the problem of assessing the relative contributions of VD and CI is solved first. At present there are no practical ways to measure BP continuously. However, there is one parameter that can be measured continuously and easily, and that is whether the trunk is positioned vertically or horizontally. If the body is still vertical when asystole starts, there is a chance that pacing might prevent syncope and falling, while there is no such chance if the body has already fallen to a horizontal position. Equipping ILR devices with position sensors would be an obvious first step to test this concept, perhaps later leading to smarter pacing.

The pathophysiological studies on nOH reveal that it is surprisingly complex, with an apparent mixture of primary defects and secondary reactions. The paradoxical occurrence of an apparent decrease in TPR in the standing position is a case in point. Studying it and its as yet unidentified mechanisms might help alleviate the orthostatic BP decrease.

## Author Contributions

JD: main concepts, outline, writing, and revising. IR: critique and writing. RT: critique and writing.

### Conflict of Interest

The authors declare that the research was conducted in the absence of any commercial or financial relationships that could be construed as a potential conflict of interest.
